# Batch Export: An automated framework for curated data extraction via the Eclipse treatment planning system

**DOI:** 10.1002/acm2.70413

**Published:** 2025-12-10

**Authors:** Ryan Truong, Lance C Moore, Casey Bojechko, Sandra M Meyers, Kelly Kisling, Xenia Ray

**Affiliations:** ^1^ Radiation Medicine and Applied Sciences University of California San Diego La Jolla California USA

**Keywords:** automation, radiation therapy, treatment planning

## Abstract

**Background:**

Deep‐learning models are useful for radiation therapy tasks such as 3D dose prediction, auto‐contouring and auto‐planning. These models require large training datasets to obtain clinically acceptable results. Currently, the process of exporting DICOM‐RT data from the Eclipse treatment planning system (TPS) is often tedious and becomes unscalable when approaching the magnitude of hundreds of patients. Thus, an efficient procedure to obtain this data would be effective for downstream research applications.

**Purpose:**

The purpose of this study was to simplify and improve the efficiency of data retrieval from the Eclipse TPS. To do so, we have created an application which exports patient treatment plans and associated images and structure sets in a parallel, streamlined process.

**Methods:**

The application was made using C# .NET and the Prism library to create a graphical user interface (GUI). EvilDICOM was integrated within the GUI to facilitate the connection to the Eclipse patient database and obtain patient plans. Data export using our application was compared to manual export using the Eclipse Export module; specifically, timing data as a function of the number of Digital Imaging and Communications in Medicine (DICOM) files exported was assessed.

**Results:**

Utilizing the application was faster than manually exporting via the TPS in cases with more than one patient. When attempting to perform an export of 20 patients’ treatment data (∼3 000 DICOM files, including the plan, structure set, dose, and all slices of the CT image), our application took 10.22 min while manual export took 22.93 min. Our application proved to be a linear‐time process and scalable to over, but not limited to, 17 000 DICOM files.

**Conclusions:**

We have developed an open‐source application to rapidly obtain patient data from Eclipse in a scalable manner. This tool addresses the challenges of manually exporting DICOM files in large magnitudes and increases the feasibility for processes like machine learning model training.

## 1 INTRODUCTION

The rise in artificial intelligence (AI) and other big data research in the fields of radiation oncology and medical physics has highlighted the need for efficient, automated tools for extracting large amounts of data. For instance, deep learning models for 3D dose prediction,^1^ auto‐contouring^2^ and auto‐planning^3^ must be trained on hundreds or even thousands of 3D images and/or plan parameters to enable accurate performance.^4^ Deep learning models in radiation therapy typically require data stored in Digital Imaging and Communications in Medicine (DICOM)^5^ format or within oncology information systems, such as Eclipse/ARIA (Varian Medical Systems, Palo Alto, CA). While these systems enable streamlined visualization of data for a given patient, the process for extracting DICOMs for external uses can be tedious. Manual export on a patient‐by‐patient basis is typically time‐consuming, infeasible, and error‐prone for the scale of AI studies. Very few published deep learning models in radiation therapy include more than 1000 patient cases,^6^ and we suspect this may be partially attributed to the difficulty in extracting datasets of this magnitude. Despite the rise in big data research in radiotherapy, there are very few published, open‐source tools for bulk Varian Eclipse DICOM data export. One open‐source framework attempting to tackle the issue is UCLHp's DicomExporter. This tool performs DICOM data export in bulk but lacks the ability to export specific plans, anonymization, and having a graphical user interface (GUI) for visualization.^7^ Other options such as Red Ion's DICOM automation tool include necessary features such as anonymization but require payment or sponsored licensing.^8^

Due to a lack of resources which show an efficient, bulk Varian ARIA database extraction, we have developed a tool to export patient DICOM data from the Eclipse TPS in batch. This tool allows users to query and export data based on patient medical record numbers, and more specifically treatment, courses, and plans. In doing so, users can identify all desired patients within a given database, view all plans for a given list of patients, and create a batch that can later be saved and used to export the associated DICOM files. Ultimately, the purpose of this study was to develop a tool for researchers and users to query, organize, and acquire patient DICOM data in an efficient manner. In doing so, challenges faced in radiation oncology and medical physics research such as data scarcity and collecting efficiency may be mitigated.

## 2 METHODS

**FIGURE 1:**
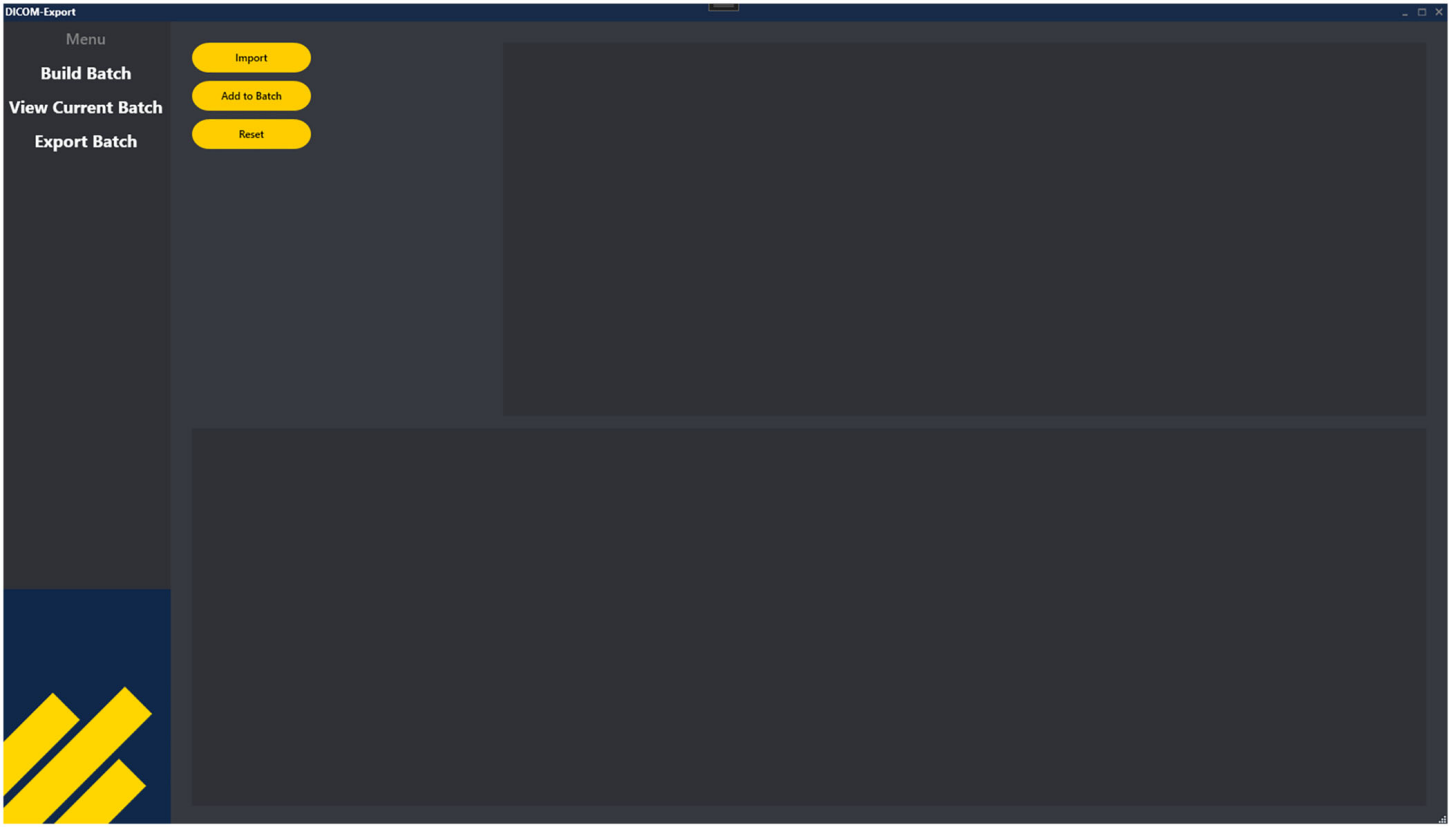
Workspace 1: *BuildBatch* ViewModel. Users import patient MRNs and, optionally, courses, and plans to be exported.

**FIGURE 2:**
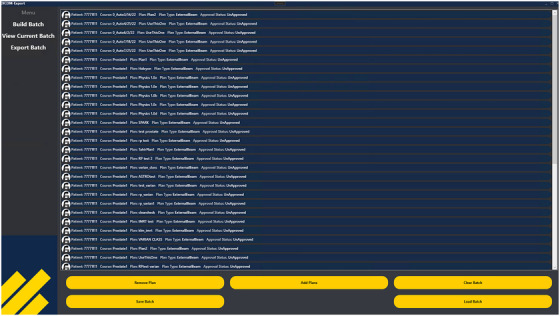
Workspace 2: *CurrentBatch* ViewModel. Users can view a list of plans here and make changes if necessary.

**FIGURE 3:**
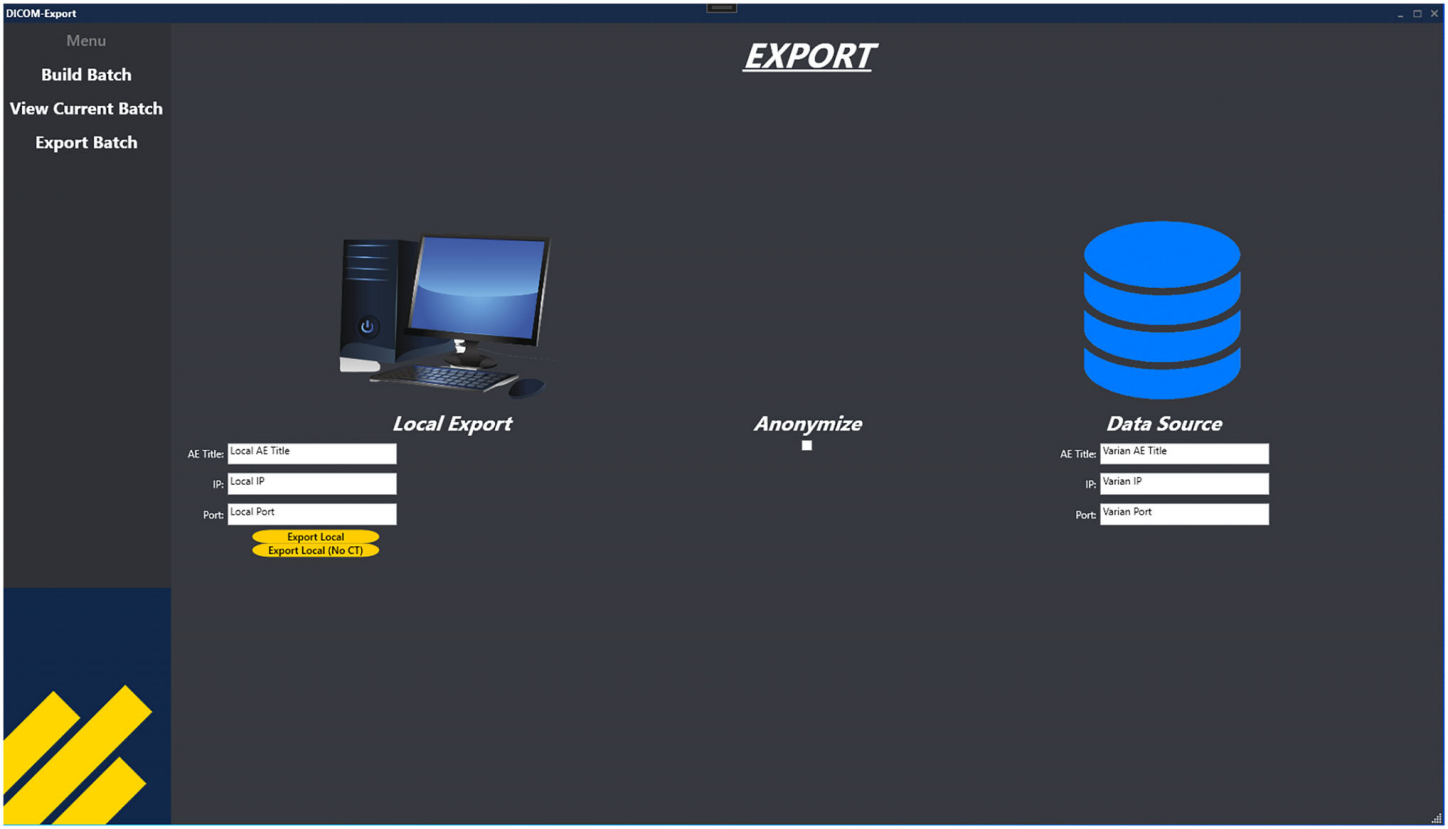
Workspace 3: *ExportBatch* ViewModel. Users configure their computer for local export and the source of the database.

The Batch Export tool is a standalone application coupled with a GUI that consists of the following three key workspaces, described in Figures 1, 2, and 3, respectively.

**Workspace 1 ‐ *BuildBatch***. *BuildBatch* is the first area of focus when a user launches the application, allowing users to select patient plans and the desired data to be exported. The user imports a list of patient medical record numbers (MRNs) from a text (txt) or comma‐separated values (csv) file, which will be queried. This file containing the MRNs must be organized in the first column and created prior to utilizing the application. All plans linked to the list of MRNs will be returned. Alternatively, the user may additionally list specific courses and plan names in their txt or csv file to pre‐filter the results. Users then choose which plans they would like to garner data from, essentially creating a “batch”.

**Workspace 2 ‐ *CurrentBatch***. *CurrentBatch* allows users to view the current batch that will be exported, make changes (e.g. add or remove plans), save the batch locally for future use, and/or finalize the list for export.

**Workspace 3 ‐ *ExportBatch***. *ExportBatch* enables data export after users have configured their local computer for export. In this module, users configure their internet protocol (IP) addresses, ports, and the source of the data, i.e. Eclipse database. Once complete, the save data folder will be selected by the user and exporting is performed.

**FIGURE 4:**
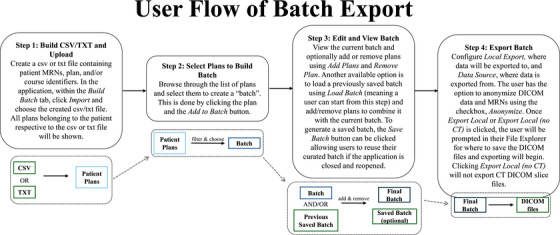
User flow of Batch Export. Users will build the batch by importing patient and/or plan identifiers, select the desired data they wish to export, edit the batch if desired, and export.

Figure 4 summarizes the user flow and walks through of the steps necessary to perform export of DICOM files.

### 2.1 Underlying technological stack and configuring the tool for use

Batch Export was created utilizing C# .NET for the front facing components. To create the GUI, Windows Presentation Foundation (WPF) was implemented via the Prism Library. Batch Export specifically is broken down into the three workspaces or ViewModels described above. The underlying mechanism to perform export was produced using a C# script and assisted with the library EvilDICOM,^9^ an open‐source C# library built for users to read and manipulate DICOM files. To obtain patient plans for viewing, Varian's Eclipse Scripting Application Programming Interface (ESAPI version 16) (Varian Medical Systems, Palo Alto, CA), specifically a Python wrapper, PyESAPI, was used. To garner these DICOM files, a proper setup of a Varian Daemon and its communication through network operations to the underlying ARIA database via EvilDICOM was necessary. These components were based on Rex Cardan's *Daemons: A tour through Varian's DICOM API* and described in more detail below.^10^

**DICOM daemon**. A DICOM daemon is an entity that exists on a network and allows for communication between the user and server. Daemons exist on the local computer and the database server to send and receive the data that is requested. To configure the daemon properly, Varian's Daemon Configuration Wizard can be used.

**Communication of daemon with Eclipse**. Built‐in network commands to control the daemons’ communication can be specified in code. For example, the functions CMOVE and CSTORE can move and store data, respectively.

With all the components established, the exporting on Batch Export requires three fields to set up the local and database daemons: application entity (AE) title, IP address, and port. The AE title is the unique name of each daemon, the IP address corresponds to the network that each daemon exists on, and the port is where communication between each daemon will occur. A visual representation of these concepts can be found in *Scripting the Varian DICOM DB Daemon with ESAPI*.^11^ Lastly, a checkbox is available for selection labeled “Anonymize”. This feature leverages Dicognito to anonymize patient MRNs and DICOM data when selected.^12^ In addition, all folder names created during export will be renamed to uncorrelated numbers removing any MRNs and course/plan names.

To successfully export data, the user will have to obtain/configure the AE title, IP addresses, and ports of both their local computer and database. Once set up, the user will be asked to input these values and can then run multiple exports within the same session without having to reinput these fields. The associated CT (one slice per CT DICOM file), RTSTRUCT, RTDOSE, and RTPLAN DICOM files will be downloaded and stored. Unlike manually exporting in Eclipse (version 16) where the user must create their own folders and organization schema, this process allows for automated organization of potentially hundreds or thousands of DICOM files by creating a hierarchical folder structure of patient > course > plan > DICOM files. To speed up the process of DICOM file retrieval, a parallel approach was implemented via the .NET Task Parallel Library. Instead of a one‐by‐one, iterative approach, the process was sped up by utilizing 75% of the available CPU threads to collect multiple DICOM files at once. The utilization of 75% was decided through experimentation as connection to the daemon frequently crashed when using 100% of available threads. Lastly, to address problems that may occur during exports, such as typos in input fields or improper setup of the application, errors are logged in a txt file within the target directory.

### 2.2 Demonstration of use

To provide a quantitative rationale and proof of efficacy for the Batch Export application, a time test trial was conducted on a research workstation (8 core 2.5 GHz CPU, 36 GB of DDR3 ram). In this trial, Batch Export was compared to manual export of patient data via the Export module in Eclipse for one, two, five, ten, and twenty test patients. The Batch Export workflow (Figure 4) included loading a patient list via a csv file containing MRNs (time to create list not included), manually selecting and exporting the desired plans. The manual export process required the following steps: (1) selecting *DICOM Import/Export*, (2) selecting the *Export* Tab, (3) selecting the export *Filter*, (4) using the *Patient Explorer* to select the desired patient, and (5) choosing the desired plan and data to be exported. Because Batch Export retrieves the CT DICOM files where one slice is one CT DICOM file, all CT image slices were also manually exported to enable a fair comparison. When performing a time test with multiple patients in Eclipse, this process was repeated for every patient. The number of DICOM files to time taken was plotted for both methods and linear regression using least squares was performed to fit the data and obtain an R‐squared value. Finally, to confirm the proposal of scalability, a larger export, not included in the table, was performed with the same 20 patients but changed to export all available plans, totaling 102 plans.

## 3 RESULTS

**FIGURE 5:**
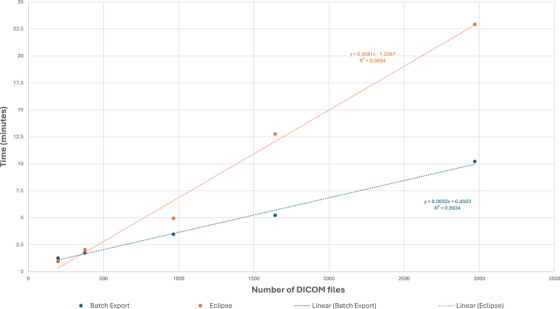
Time for manual and Batch Export versus number of DICOM files exported. Digital Imaging and Communications in Medicine, DICOM.

The time trials demonstrated that Batch Export is faster than manual Eclipse export in all cases, except for the export of a single patient dataset (see Table 1 and Figure 5).

**TABLE 1:** DICOM file export time analysis for manual export via Eclipse compared to Batch Export.

		Time (min)	
Patient(s)	Number of DICOM files	Eclipse (manual export)	Batch Export
1	197	0.95*	1.27
2	376	2.05	1.75*
5	965	4.95	3.47*
10	1,642	12.77	5.23*
20	2,970	22.93	10.22*

* Indicates which time (Eclipse or Batch Export) was shorter.

Abbrevation: Digital Imaging and Communications in Medicine, DICOM

Batch Export was slower for a single patient due to the time required to input the AE title, IP, and port. For accuracy and fairness in timing, each trial was considered a new session, which entailed entering the required prerequisites. However, the improved efficiency with Batch Export became more apparent with increasing DICOM files. Manually exporting via Eclipse was found to be a linear‐timed process, as each patient took about one minute to set up and export ∼200 DICOM files (about 0.37 s per DICOM file, on average). While Batch Export also followed a linear trend, the slope of DICOM files to time was about half as steep (approximately 0.26 s per DICOM file), meaning Batch Export took about half the time to download the same amount of DICOM files. Performing the larger export of 17488 DICOM files, garnered from 20 patients (102 total plans) took 39.55 min (approximately 0.14 s per DICOM file). Given the export rate decreased compared to the rate observed in the smaller time trial, this provides evidence for consistency in results for larger scale and multi‐plan projects.

## 4 DISCUSSION

With the growing need to access patient data in bulk for research, Batch Export serves as a tool to simplify the process and efficiently garner DICOM files. Manually exporting patient‐by‐patient in Eclipse requires constant user intervention and becomes impractical when even hundreds let alone thousands of patients need to be exported. Batch Export allows the user to import a list of patients and either manually select the desired plans within the application or import a list of plans. While the export configuration and plan selection process require some up‐front time, this time becomes more negligible as the number of DICOMs exported increases.

The underlying mechanism of Batch Export relies on the use of EvilDICOM and ESAPI (PyESAPI) to connect to the patient database. While we report the time required for various sized batches, the exact speed of Batch Export in other clinical settings will be dependent on the computational power available. Similarly, dissemination of Batch Exports will be limited to clinics with a thick‐client workstation which has access to Eclipse and ARIA database rights, ESAPI, Visual Studio, and Python/Dicognito, as all these tools will be necessary to run the application.

We have made Batch Export available on the Varian Medical Affairs GitHub for download. The proper configuration of the application, specifically locating the correct IP addresses, ports, and registration of the workstation for the Varian DB Daemon, may require help from the local user's IT department. As a layer of security, the DB Daemon configuration requires the AE titles used in Batch Export to match what was registered.

Our Batch Export tool has several limitations to be addressed and updated in future versions. Filters and options will be added in future versions to help automate the curation and give the user more control over the data exported. For example, we plan to incorporate an option to query patient data within Eclipse based on features such as treatment site (e.g. “Prostate and Nodes”), modality (e.g., “IMRT” or “VMAT”) and plan approval status (e.g., “Treatment approved”). Another update planned for further efficiency is automated creation and loading of a config file for the AE titles, IP addresses, and port numbers after first input. This will prevent users from having to re‐enter information if the application is closed. Lastly, more thorough anonymization, specifically a comprehensive folder naming schema and master key to un‐anonymize is expected in the following version.

Despite these issues, this tool has been very useful for numerous deep learning projects at our institution, including 3D dose prediction for cervical brachytherapy and breast radiotherapy,^1^, ^3^ classifying patient‐shifts during treatment using electronic portal imaging devices (EPID) images,^13^ and assembling one of the largest datasets for 3D dose prediction.^6^

## 5 CONCLUSIONS

We have developed an open‐source application to rapidly obtain patient data from Eclipse in a scalable manner. The tool provides significant benefits over manual Eclipse export, enabling large DICOM datasets to be exported and organized with minimal user intervention. Batch Export is exclusive to ARIA/Eclipse frameworks and other tools may exist for different treatment planning systems. However, given the prevalence of this oncology information system worldwide and the current lack of effective tools for automated DICOM export within Eclipse, we felt that publishing on this methodology and making our tool open source would benefit many institutions. We believe that this tool could help to solve a recurring bottleneck in medical physics research, facilitating more streamlined model development and evaluation.

## AUTHOR CONTRIBUTION

Ryan Truong conducted the experiments, edited the code and wrote the manuscript. Lance Moore created and tested the code and provided feedback and direction. Casey Bojechko created the initial draft of the manuscript. Casey Bojechko and Sandra Meyers facilitated the planning and direction for the project. Kelly Kisling and Xenia Ray provided feedback and insight for the manuscript.

## CONFLICT OF INTEREST STATEMENT

The authors have no relevant conflicts of interest to disclose.
